# The reporting of studies using routinely collected health data was often insufficient

**DOI:** 10.1016/j.jclinepi.2016.06.005

**Published:** 2016-11

**Authors:** Lars G. Hemkens, Eric I. Benchimol, Sinéad M. Langan, Matthias Briel, Benjamin Kasenda, Jean-Marie Januel, Emily Herrett, Erik von Elm

**Affiliations:** a Department of Clinical Research, Basel Institute for Clinical Epidemiology and Biostatistics, University Hospital Basel, Spitalstrasse 12, CH-4031 Basel, Switzerland; b Department of Pediatrics, Children's Hospital of Eastern Ontario, School of Epidemiology, Public Health and Preventive Medicine, University of Ottawa, 401 Smyth Road, Ottawa, ON K1H 8L1, Canada; c Institute for Clinical Evaluative Sciences, G1 06, 2075 Bayview Avenue, Toronto, ON M4N 3M5, Canada; d Department of Non-communicable Disease Epidemiology, Faculty of Epidemiology and Population Health, London School of Hygiene and Tropical Medicine, Keppel Street, London, WC1E 7HT, United Kingdom; e Department of Clinical Epidemiology and Biostatistics, McMaster University, 1280 Main Street West, Hamilton, ON L8S 4K1, Canada; f University Institute of Higher Education and Research in Health Care (IUFRS), Faculty of Biology and Medicine, University of Lausanne, Biopole 2, Route de la Corniche 10, CH-1010, Lausanne, Switzerland; g Cochrane Switzerland, Institute of Social and Preventive Medicine, Lausanne University Hospital, Route de la Corniche 10, CH-1010, Lausanne, Switzerland

**Keywords:** Routinely collected data, Observational studies, Research reporting, Guidelines, Research design, Bibliometrics

## Abstract

**Objectives:**

To assess reporting quality of studies using routinely collected health data (RCD) to inform the REporting of studies Conducted using Observational Routinely collected health Data (RECORD) guideline development.

**Study Design and Setting:**

PubMed search for observational studies using RCD on any epidemiologic or clinical topic. Sample of studies published in 2012. Evaluation of five items based on the STrengthening the Reporting of OBservational studies in Epidemiology (STROBE) guideline and eight newly developed items for RCD studies.

**Results:**

Of 124 included studies, 39 (31.5%) clearly described its design in title or abstract. Complete information to frame a focused research question, that is, on the population, intervention/exposure, and outcome, was provided for 51 studies (41.1%). In 44 studies where definitions of codes or classification algorithms would be necessary to operationalize such a research question, only nine (20.5%) reported all items adequately. In 81 studies describing multivariable analyses, 54 (66.7%) reported all variables used for modeling and 34 (42.0%) reported basic details required for replication. Database linkage was reported adequately in 12 of 41 studies (29.3%). Statements about data sharing/availability were rare (5/124; 4%).

**Conclusion:**

Most RCD studies are insufficiently reported. Specific reporting guidelines and more awareness and education on their use are urgently needed.

**What is new?**

**Key findings**
• Most studies using routinely collected health data (RCD) are insufficiently reported. For example, it is frequently impossible to know which exposure or intervention is associated with which outcome in which population or minimal prerequisites for replication/assessment of scientific validity are often lacking.

**What this adds to what was known?**
• Even years after introducing reporting guidelines for observational studies, many studies from various clinical and epidemiologic areas are poorly reported.

**What is the implication and what should change now?**
• Specific reporting guidelines for studies using RCD are necessary to address specific characteristics of such research.
• Authors, peer reviewers, and editors need training to apply both novel and established reporting guidelines to ensure better and more complete research reporting.

## 1 Introduction

Routinely collected health data (RCD) are defined as data collected for purposes other than research [1], [2]. Examples include health administrative data, electronic health records, and disease or clinical registries. Increased ability to store, process, and quickly access large amounts of such data led to increasing collection and usage for health research. Using such novel data sources involves unique challenges for research reporting, for example, the description of database characteristics or record linkage methodology [3]. Poor reporting wastes efforts and resources [4]. Guidelines such as the STrengthening the Reporting of OBservational studies in Epidemiology (STROBE) statement have been developed and endorsed by many journals to improve reporting of biomedical research [5]. Inadequate or incomplete reporting has been shown in observational studies on general medical interventions that were published before introduction of STROBE [6] and in more recent evaluations that addressed specific research areas, including cancer [7], hand surgery [8], dermatology [9], plastic surgery [10], or magnetic resonance imaging [11].

We analyzed the reporting of any type of observational study using RCD in a randomly selected sample of studies published in 2012 which were identified in PubMed. We focused on reporting domains that are central with regard to the study's design, its research question, and basic prerequisites for study results replication. We selected items addressing these reporting domains in STROBE, and we developed a set of new items deemed specifically important for reporting of research using RCD. This new set included items that directly correspond to the selected STROBE items and items that focus on selected specific characteristics of RCD research.

Using a sample of recent publications, we systematically evaluated these reporting items. In ancillary analyses, we explored if reporting affects both low- and high-impact journals and if better reporting is associated with more citations.

We aimed to assess the present state of reporting and provide a first empirical estimate of its quality to inform the development of a specific reporting guideline for RCD studies by the REporting of studies Conducted using Observational Routinely collected health Data (RECORD) working committee [12]. RECORD has recently been published as an extension of the STROBE guidelines and aims to enhance transparency of research reporting and provide guidance to adequately report methods and findings of research using RCD [13].

## 2 Methods

### 2.1 Eligibility of studies

We selected a sample of English language studies that used RCD and reported outcomes related to the health status of persons or a population, such as mortality or morbidity. For example, we included publications detailing epidemiologic research on incidence and prevalence of diseases or risk factors or comparative effectiveness research studies measuring treatment effects. We did not consider studies on outcomes such as costs or care processes. We included nonexperimental studies in humans based on any type of health data that were routinely collected, that is, for purposes other than research. We also included analyses based on registries, albeit registries characteristically comprise at least one actively collected data element [14]. There were no restrictions with respect to characteristics of study participants.

### 2.2 Literature search

We searched PubMed for studies published in 2012 (search date June 6, 2013) using terms related to RCD, including constructs for “database,” “registries,” “electronic health records,” and “administrative data/routine data” (Webappendix 1 at www.jclinepi.com). We integrated the search strategy for electronic health records provided by the National Library of Medicine into our strategy [15]. An information specialist formally peer reviewed the strategy [16].

### 2.3 Study selection

The 24,929 hits in PubMed were exported to Microsoft Excel (Microsoft Corporation, Redmond, WA, USA) and ranked in random order. Two independent reviewers (L.G.H. and one of E.I.B., S.M.L., E.v.E.) screened titles and abstracts in this order and excluded studies obviously not meeting eligibility criteria. Any disagreements were resolved by discussion. We obtained full texts of the first 150 potentially eligible references. The sample size was determined arbitrarily based on our experience with similar projects [9]. We determined eligibility of full texts in teams of independent reviewers (two of L.G.H., E.I.B., S.M.L., M.B., B.K., J-.M.J., E.H., E.v.E.). Any disagreements were resolved by consensus among the larger group.

### 2.4 Extraction of study characteristics

For each eligible RCD study, we extracted the study characteristics including RCD type, area of research (epidemiology, e.g., risk factors of diseases; clinical/medical, e.g., comparative effectiveness of medical treatments), type of disease/condition of participants, and characteristics of reported analyses. We classified types of RCD as shown in Webappendix 2 at www.jclinepi.com. Data were extracted by teams of two reviewers (two of L.G.H., E.I.B., S.M.L., M.B., B.K., J-.M.J., E.H., E.v.E.). Any disagreements were resolved by consensus among the two reviewers or with a third reviewer (L.G.H.).

One reviewer recorded if journals endorsed the STROBE statement according to information on the STROBE website (L.G.H.) [17].

To compare the citation impact metrics of adequately and inadequately reported studies (per reporting item), bibliographic information was extracted from ISI Web of Knowledge, that is, the 2012 impact factor (IF) of the publishing journal and the number of citations (all databases) up to February 2015 that a study has received. This was extracted by one researcher and verified by another.

### 2.5 Evaluation of reporting quality

#### 2.5.1 Selection and development of reporting items

We evaluated five selected reporting items based on the STROBE checklist [5] (S1–S5) and eight newly developed items (R1–R8) for RCD research (Table 1; the prefix “S” denotes items based on STROBE; the prefix “R” denotes newly developed items deemed important specifically for RCD studies. Further details and examples of reporting are shown in Webappendix 3 at www.jclinepi.com)

We aimed to reflect the reporting of the study design and research question, study replication, and RCD specifics. Selection of STROBE items and development of new RCD items was based on expert opinion of the authors (L.G.H., E.I.B., S.M.L., E.v.E.) without a formalized development process.

We operationalized all items into dedicated questions that can be clearly answered with “yes,” “partly,” or “no,” indicating adequate (“yes”) or inadequate (“no”) reporting. We used the “partly” answer when not all aspects were adequately reported, for example, when several eligibility criteria existed, but some were described and others were not. We accepted references to other publications as adequate descriptions.

We tested the item operationalization and developed rules and detailed extraction instructions in a small pilot study using a selected sample of three articles that were extracted and assessed by the larger group of the authors. We then assessed 40% of the 150 full texts and formally calibrated extractions of eligible articles among reviewers. We clarified the operationalization of the reporting items by specifying the wording of extraction instructions before we completed our extractions for the remaining 60% of the sample.

#### 2.5.2 Reporting items

First, we evaluated if the study title and abstract allowed a basic classification of the design of the study and indicated the use of routine data (S1 and R1).

Second, we assessed if sufficient details of the evaluated population, exposures or interventions (or risk factors, predictors, effect modifiers, and so forth), and outcomes were reported (S2–S4). Transparent reporting of these items is crucial for research translation to health care, and specifically in a medical context, this information would allow framing of a focused PICO question on the medical problem (PICO: Population, Intervention, Control, Outcome) [19].

Third, we assessed codes and classification algorithms and other basic requirements for replication of analyses (R2–R5, S5a, S5b). We assessed if sufficient details were reported on the population, intervention/exposure, and outcomes (R2–R4) as prerequisite to facilitate repetition of the analyses in the same or another data set (for simplicity, we do not differentiate between replication and reproduction). RCD studies typically require an exact operationalization of the same items that are used for framing a focused research question in the previous domain. For example, when administrative data such as diagnostic codes are used to define a population with type 2 diabetes, this would require an exhaustive list of all codes used to define this disease in the specific study context (e.g., International Classification of Diseases E11); when electronic medical records are used to identify these patients, a list of specific terms indicating the disease would be required (e.g., “diabetes mellitus type 2” or “T2DM”). We evaluated these code-related items (i.e., R2–R4) only for studies using administrative data or electronic health or medical records because in this context, the retrospective identification of the population, exposures or intervention, or outcomes usually requires such codes and/or classification algorithms. In studies using registry data, codes may be less relevant, for example, when patients are actively recruited or outcomes are specifically measured for the purpose of the registry. We also evaluated whether the description of the analyzed databases was clear enough to assess the generalizability of the results and to replicate the findings in other contexts (R5). Then, we evaluated the description of variables and models in statistical analyses (S5a, S5b), but we only assessed multivariable analyses as they are the most frequently used statistical method in this research field.

Fourth, we evaluated RCD specifics (R6–R8), that is, the reporting of methods for linkage of multiple databases (where applicable); any statements about data sharing issues or the availability of the used data set for other researchers; and any statements about the validation of coding or classification algorithms used for identification of patients, interventions or exposures, or outcomes were made. We analyzed this item in all studies using electronic health or medical records or administrative data for the reasons outlined above (items R2–R4).

### 2.6 Statistical analysis

Citation metrics of studies with adequate and inadequate reporting were compared per reporting item using the Mann–Whitney U test. We used Stata 13.1 (Stata Corp, College Station, TX, USA) for statistical analysis. *P*-values are two tailed.

## 3 Results

### 3.1 Selection and characteristics of studies

Of 150 articles evaluated as full texts, 26 articles were excluded (21 were no RCD studies and 5 reported no health-related outcomes). We included 124 eligible articles for further analysis. Most studies used registry data (*n* = 70; 56.5%) or administrative health data (*n* = 40; 32.3%) (Table 2). A single data source was used in 74 studies (59.7%) and two data sources in 24 studies (19.4%). Epidemiologic questions were addressed in 91 studies (73.4%) and clinical questions in 25 studies (20.2%). Most studies reported multivariable models (*n* = 81; 65.3%) and five studies (4.0%) used propensity scores.

### 3.2 Reporting quality

The study design was not clearly described in the title or abstract of most studies (inadequate reporting for S1: 62.9%), but many studies were there clearly described as using RCD (adequate reporting for R1: 71.8%; Table 3).

Many studies clearly reported the evaluated population, exposures or interventions, or outcomes according to STROBE (adequate reporting for S2: 74.2%; S3: 77.5%; S4: 75.7%; Table 3), but only 51 studies (41.1%) reported all of the three items adequately.

Most studies did not adequately report the coding or classification algorithm (inadequate reporting for R2: 53.2%; R3: 58.2%; R4: 42.6%; Table 3). In 44 studies, replication would require coding or classification information for all three items because they used administrative data or electronic medical/health records to describe the population, exposures or interventions, and outcomes. Only 9 of these 44 studies (20.5%) reported all three items adequately, whereas 17 (38.6%) reported all items inadequately, as would be required to frame a focused research question.

Across 81 studies using multivariable analyses, 54 studies provided a complete list of used variables (S5a adequate: 66.7%), but only 34 studies sufficiently reported basic details required for replication (S5b adequate: 42%).

The analyzed databases were clearly described for most studies (R5 adequate: 60.5%), but the majority of studies did not clearly report the methods used for database linkage (R6 inadequate 68.3%), did not make statements on data sharing or availability of data sets (R7 inadequate: 96%), or about the validation of classification algorithms (R8 inadequate: 75.8%).

The agreement between both reviewers across all 14 items was 74.1% (median agreement per item).

### 3.3 Association with journal IF and citation count

The journal IF was higher for studies that clearly reported details on the statistical analyses (S5a: IF 3.5 vs. 3.2; *P* = 0.027. S5b: IF 3.7 vs. 3.2, *P* = 0.013), provided sufficient details on the study outcomes (S4: IF 3.4 vs. 2.6; *P* = 0.047), and clearly described the coding and classification of participants (R2: IF 3.6 vs. 2.5; *P* = 0.006) (Table 4). We found no significant association between journal IF and other reporting domains or between reporting quality and number of citations.

## 4 Discussion

Our systematic analysis of 124 studies reveals a number of deficiencies in the reporting of research using RCD. Most studies were insufficiently reported as they have substantial reporting deficits specifically concerning their particular methodology for using RCD (e.g., database linkage or used codes and their accuracy and validity). This underlines the necessity to establish specific reporting guidelines for RCD studies, such as the REporting of studies Conducted using Observational Routinely collected health Data (RECORD) statement [13] and the importance of adequate implementation by journals, peer reviewers, and funding agencies.

We also found substantial reporting deficits that concern observational studies in general. They relate to areas of reporting that are already addressed by established reporting guidelines (STROBE). We found that less than half of the evaluated studies provided complete information to frame a focused research question, that is, it was frequently impossible to know which exposure or intervention was associated with which outcome and in which population. The descriptions of statistical analyses in most studies lacked minimal prerequisites for replication and assessment of scientific validity: about one-third of studies that used multivariable models did not provide a complete list of the variables used for modeling; basic details on how the variables were used were provided in less than half of the studies.

Journal IF was associated with quality of reporting in few areas, underlining that insufficient reporting is a ubiquitous problem and affects both low- and high-impact journals. We found no relationship between reporting and citation counts, in contrast to a recent study that evaluated reporting of systematic reviews and meta-analyses in one medical field (radiology) [20]. Since only few articles were published in journals endorsing STROBE, we did not evaluate the association of STROBE endorsement and reporting quality. A recent Lancet series corroborated the fact that poor reporting of key information is endemic in various areas of health research and affects any study type, including randomized trials, observational studies, laboratory research, and animal studies [4]. Our analysis is consistent with previous work on other forms of observational studies [6], [7], [8], [9], [10], [11]. Even years after publication of the STROBE guideline, reporting of observational research is still deficient.

Our work has some limitations. First, we explored only a small number of reporting items that reflect information which we deemed essential for RCD studies and there are further and more expanded items in the RECORD guideline [13]. Other relevant issues remain unaddressed in our analysis. For example, when we evaluated the replicability of statistical analyses, we addressed only obvious aspects of reporting of multivariable analyses. According to the International Committee of Medical Journal Editors, statistical methods should be described “with enough detail to enable a knowledgeable reader with access to the original data to judge its appropriateness for the study and to verify the reported results” [21]. We did not evaluate other details that a reader would likely require for replication, such as the availability of statistical code, but we found that almost all articles lacked a statement about access to the original data.

Second, our sample drawn from publications of the year 2012 in English language from journals indexed in MEDLINE is only a fraction of the entire literature. However, it is unlikely that reporting quality has substantially changed in the meantime and it remains speculative if our findings also apply to studies reported in other journals or languages.

Third, we searched the literature using terms that might have enriched the sample with studies mentioning RCD terms in title or abstract. Thus, the finding that titles or abstracts of RCD studies often indicate the use of routine data might be overly optimistic. However, we used a peer-reviewed search strategy that was deemed accurate and complete for the identification of observational RCD studies. Therefore, we believe the sample of included studies allows generalizing the findings to the larger RCD literature.

Fourth, we assessed the reporting by two independent reviewers using their best subjective judgment. However, all involved researchers had training in research reporting, used standardized and piloted electronic extraction forms with detailed instructions, and we systematically calibrated our extractions during the process resulting in an overall interreviewer agreement of 74.1% across all items. The experience from the consensus process ultimately informed the discussion and item operationalization during the RECORD guideline development.

Finally, modern database analyses may frequently use study designs that do not clearly fit into traditional study design classifications such as “cohort study” or “case–control study.” Authors of such research may find the STROBE recommendation to “indicate the study's design with a commonly used term” as inappropriate. This issue should be considered in future versions of the reporting guidelines. Our finding with respect to reporting of the study design should not be overrated and cautiously interpreted.

Our results suggest that poor reporting of key study information is prominent in RCD research and may limit its further use, for example, by limiting the assessment of its scientific validity or hindering its replication. The incomplete or imprecise description of research questions in most studies may waste research resources, for example, by unnecessary replication efforts or misguided funding decisions in their research fields [4]. Generally, reporting deficits may lead to inefficient, misguided, or haphazard translation of research findings to public health actions or medical care.

This study provides a benchmark for the reporting quality of RCD studies. Preliminary findings of this project were presented to the working committee of RECORD and informed the guideline development. Areas discovered by this study to be poorly reported have been emphasized in the RECORD guideline [13]. Authors, peer reviewers, and editors need training to apply both novel and established reporting guidelines to ensure better and more complete research reporting. We believe that adoption of such guidelines and education on their use is particularly urgent to improve the utility of research using RCD.

## Figures and Tables

**Table 1:** Items for evaluation of reporting quality

Item	Description
[S1]	Is the study's design indicated with a commonly used term in the title or the abstract? We accepted any term for study designs (such as “cohort study” or “case–control study”) used in typical study classification schemes [18].
[R1]	Is the use of routinely collected data or registry data clearly mentioned in the title or the abstract using common terms? We evaluated whether information in the title or abstract allows a reader or a database search engine to clearly recognize the use of routinely collected or registry data.
[S2]	Are the selection criteria for the analyzed participants clearly described? This was deemed adequate when the study participant selection was reported in a way that it would be clear to whom the results directly apply and for whom they would not be applicable.
[R2]	Is the coding/classification of patients clearly described with sufficient details? We deemed reporting adequate when the description of the coding or classification algorithm was sufficiently clear to allow replication of the analysis.
[S3]	Are all interventions/exposures of interest clearly described? We deemed an exposure or intervention (or risk factor, predictor, effect modifier, and so forth) sufficiently described when the provided details would allow the application of the intervention or the measurement of exposure (or risk factor and so forth) in practice. The reader should know precisely which action (e.g., prescription of a certain dose of a drug) or exposure is being assessed in the study [19].
[R3]	Is the coding/classification of the interventions/exposures clearly described with sufficient details? We deemed reporting adequate when the description of the coding or classification algorithm was sufficiently clear to allow replication of the analysis.
[S4]	Are all outcomes of interest clearly described? The outcome description was deemed adequate if it was equivalent to an outcome description in a planned prospective study designed to specifically investigate the issue (regardless whether such study would be interventional or observational, feasible or not) and if the detail given was sufficient for others to replicate the study. We did not assess if broad or specific outcomes were used, but we assessed if the reporting clearly defined the outcome and how it was measured and defined. For example, we deemed it insufficient when authors reported “we analyzed effects on hypertension” without giving a definition of hypertension (e.g., defined by more than one prescription of an antihypertensive drug within 6 months); or when authors say “we evaluated effects on mortality” without stating whether all-cause or cause-specific mortality has been investigated and without reporting the time-frame (e.g., in-hospital mortality or 30-day-after discharge mortality).
[R4]	Is the coding/classification of the outcomes clearly described with sufficient details? We deemed reporting adequate when the description of the coding or classification algorithm was sufficiently clear to allow replication of the analysis. We deemed it unnecessary for replication that all-cause mortality is operationalized with a specific code because this outcome is typically clear.
[S5]	Are the independent variables in analytic models (1) listed (or are the strategies used to create models reported)? We deemed reporting adequate when all analyzed variables (e.g., age, body weight, smoking) were listed. (2) described in sufficient detail (including categorization) to replicate the study? We deemed reporting adequate when details were provided on how the variables were included in the statistical models (e.g., age and body weight both as continuous variable and smoking as categorical variable such as “never smokers,” “previous smokers,” “smoking 1 to 10 cigarettes daily,” and “smoking more than 11 cigarettes daily”).
[R5]	Are the characteristics of the analyzed data sets clearly described, including (1) covered time period, (2) location, (3) setting, and other potentially important factors? We deemed that reporting was adequate when the covered time period, geographic location, care setting, and other potentially important factors (e.g., essential details about type of data used; decision on a case-by-case basis) were reported.
[R6]	Are the methods of linkage of databases clearly described (if applicable)?
[R7]	Are issues of data sharing clearly addressed, i.e., whether the data set is publicly available (or shared on request)? We accepted any statement regardless of how detailed it was.
[R8]	Is the validation of classification algorithms used for patients, interventions/outcomes/exposures described (if applicable)?

**Table 2:** Characteristics of analyzed RCD study sample

Characteristics	Studies, *n* (%)
Studies (*n*)	124 (100)
Number of routine data sources	
Single data source	74 (59.7)
2 data sources	24 (19.4)
3 data sources	9 (7.3)
4 data sources	9 (7.3)
5 or more data sources	8 (6.5)
Type of routine data^a^	
Administrative data, not health	26 (21.0)
Administrative health data	40 (32.3)
Prescription data	13 (10.5)
Other administrative health data	37 (29.8)
EMR/EHR	19 (15.3)
Registry	70 (56.5)
Disease registry	64 (51.6)
Device registry	7 (5.6)
Other	14 (12.1)
Area of research	
Clinical/medical	25 (20.2)
Epidemiology	91 (73.4)
Other or both areas	8 (6.5)
Statistical analyses	
Multivariable analyses^a^	81 (65.3)
Propensity scores^a^	5 (4.0)
Other or purely descriptive	43 (34.7)
Type of condition	
Cancer	35 (28.2)
Cardiovascular disease	17 (13.7)
Endocrinology	6 (4.8)
Nephrology	3 (2.4)
Neurology/psychiatry	10 (8.1)
Other or healthy participants	53 (42.7)
Citation impact	
IF 2012 (median, IQR) (range), *n* = 118	3.12 (2.16; 4.16) [0.15–25.12]
Citations (median, IQR) (range), *n* = 124	5 (2; 10.5) [0–68]
Endorsement of STROBE	17 (13.7)

*Abbreviations:* STROBE, STrengthening the Reporting of OBservational studies in Epidemiology; RCD, routinely collected data; EMR, electronic medical record; EHR, electronic health record; IF, impact factor; IQR, interquartile range.

Citations up to February 2015 as of ISI Web of Knowledge (all databases); STROBE endorsement as of March 2016.

a More than one category may apply.

**Table 3:** Reporting quality in RCD studies

Reporting item	Reporting adequate			Studies, *n*	Interrater agreement (%)
	Yes, *n* (%)	Partly, *n* (%)	No, *n* (%)		
STROBE related					
[S1] Study design in title or abstract	39 (31.5)	7 (5.6)	78 (62.9)	124	73.1
[S2] Selection criteria of participants	92 (74.2)	18 (14.5)	14 (11.3)	124	77.6
[S3] Details on interventions/exposures	86 (77.5)	13 (11.7)	12 (10.8)	111	69.2
[S4] Details on outcomes	87 (75.7)	19 (16.5)	9 (7.8)	115	78.5
[S5a] Variables used for analyses listed^a^	54 (66.7)	15 (18.5)	12 (14.8)	81	66.2
[S5b] … described in sufficient detail	34 (42)	10 (12.3)	37 (45.7)	81	50.6
RCD related					
[R1] Use of RCD/registry data in title/abstract	89 (71.8)	1 (0.8)	34 (27.4)	124	75.2
[R2] Coding of participants	26 (41.9)	3 (4.8)	33 (53.2)	62	74.1
[R3] Coding of interventions/exposures	20 (36.4)	3 (5.5)	32 (58.2)	55	57.7
[R4] Coding of outcomes	29 (53.7)	2 (3.7)	23 (42.6)	54	74.0
[R5] Characteristics of data source	75 (60.5)	32 (25.8)	17 (13.7)	124	73.0
[R6] Methods of data linkage	12 (29.3)	1 (2.4)	28 (68.3)	41	92.3
[R7] Data availability/sharing	3 (2.4)	2 (1.6)	119 (96)	124	87.8
[R8] Validation of classification algorithms	13 (19.7)	3 (4.6)	50 (75.8)	66	82.3

*Abbreviations:* STROBE, STrengthening the Reporting of OBservational studies in Epidemiology; RCD, routinely collected data.

Not all items were applicable for all studies, please see Section 2 for details.

a Or the strategies used to create models reported.

**Table 4:** Association of reporting quality with journal impact factor and citation count

Reporting item	Reporting adequate	Impact factor 2012	*P*-value	Citations per article	*P*-value	Studies (*n*)
STROBE related						
[S1] Study design in title or abstract	Yes	3.4 (1.9; 4.5)	0.87	5 (2; 11)	0.427	39
	No	3 (2.3; 3.9)		4 (2; 9)		78
[S2] Selection criteria of participants	Yes	3.1 (2.1; 4.5)	0.737	5 (2; 9.5)	0.973	92
	No	3.2 (2.8; 3.9)		7 (4; 15)		14
[S3] Details on interventions/exposures	Yes	3.3 (2.2; 4.3)	0.733	5 (2; 11)	0.268	86
	No	3.1 (2.5; 3.2)		3 (1; 9.5)		12
[S4] Details on outcomes	Yes	3.4 (2.2; 4.5)	0.047	5 (2; 11)	0.057	87
	No	2.6 (1.9; 3.4)		4 (1; 10)		9
[S5a] Variables used for analyses listed^a^	Yes	3.5 (2.2; 4.9)	0.027	5 (2; 12)	0.223	54
	No	3.2 (2.6; 4.2)		4.5 (2; 13)		12
[S5b] … described in sufficient detail	Yes	3.7 (2.4; 5.1)	0.013	6 (3; 11)	0.277	34
	No	3.2 (2.2; 4.2)		6 (2; 13)		37
RCD related						
[R1] Use of RCD/registry data in title/abstract	Yes	3.3 (2.1; 4.4)	0.554	5 (2; 10)	0.876	89
	No	2.9 (2.3; 3.4)		3.5 (2; 9)		34
[R2] Coding of participants	Yes	3.6 (2.8; 5.6)	0.006	5.5 (3; 11)	0.427	26
	No	2.5 (1.9; 3.7)		2 (1; 7)		33
[R3] Coding of interventions/exposures	Yes	3.5 (2.3; 4.7)	0.081	5.5 (2.5; 15)	0.259	20
	No	2.3 (1.9; 3.7)		3 (1; 7)		32
[R4] Coding of outcomes	Yes	3.6 (2.2; 5.2)	0.11	5 (3; 7)	0.882	29
	No	2.6 (2.2; 3.3)		3 (1; 11)		23
[R5] Characteristics of data source	Yes	3.1 (2.2; 4.3)	0.538	4 (2; 11)	0.471	75
	No	2.8 (2.2; 3.7)		6 (1; 11)		17
[R6] Methods of data linkage	Yes	2.8 (2.1; 4.8)	0.963	4.5 (3; 10)		12
	No	3.3 (2.1; 4.5)		5 (2; 10)	0.932	28
[R7] Data availability/sharing	Yes	17.2 (2.4; 18)	0.108	14 (4; 19)	0.135	3
	No	3.1 (2.2; 4.1)		4 (2; 10)		119
[R8] Validation of classification algorithms	Yes	3.5 (2.5; 5.4)	0.206	6 (4; 8)	0.466	13
	No	2.7 (2.2; 4.2)		3.5 (1; 8)		50

*Abbreviations:* STROBE, STrengthening the Reporting of OBservational studies in Epidemiology; RCD, routinely collected data.

Impact factors for 2012 were not available for publishing journals of six studies; citations up to February 2015 as of ISI Web of Knowledge (all databases); impact factors and citation counts are medians with interquartile ranges.

a Or the strategies used to create models reported.

## Supplementary data

Webappendices 1–3
