# Supplementary material for: The reporting of studies using routinely collected health data was often insufficient
Source: J Clin Epidemiol. 2016 Nov;79:104–11. doi: 10.1016/j.jclinepi.2016.06.005 (PMC5152936; doi:10.1016/j.jclinepi.2016.06.005)
Supplement: Webappendices 1–3 [file mmc1.pdf]

Webappendix 1: Search Strategy (PubMed)

Search	Query
#11	(#10 AND ("2012/01/01"[PDAT] : "2012/12/31"[PDAT]))
#10	(#9 NOT #8)
#9	(#5 AND #6 AND #7)
#8	(Review[ptyp] OR Addresses[ptyp] OR Biography[ptyp] OR Bibliography[ptyp] OR Autobiography[ptyp] OR Case Reports[ptyp] OR Clinical Conference[ptyp] OR Comment[ptyp] OR Congresses[ptyp] OR Consensus Development Conference[ptyp] OR Consensus Development Conference, NIH[ptyp] OR Editorial[ptyp] OR Letter[ptyp] OR Dictionary[ptyp] OR Directory[ptyp] OR Historical Article[ptyp] OR Legal Cases[ptyp] OR Meta-Analysis[ptyp] OR Guideline[ptyp] OR News[ptyp] OR Newspaper Article[ptyp] OR Patient Education Handout[ptyp] OR Personal Narratives[ptyp] OR Practice Guideline[ptyp] OR Interview[ptyp] OR In Vitro[ptyp] OR Legislation[ptyp] OR Lectures[ptyp] OR Video-Audio Media[ptyp] OR Webcasts[ptyp] OR Portraits[ptyp])
#7	English[lang]
#6	"humans"[mh]
#5	(#1 OR #2 OR #3 OR #4)
#4	(Administrative[tiab] OR Claims[tiab] OR “routine data”[tiab] OR “routinely collected”[tiab])
#3	(((health information exchange[tw] OR hie[tw] OR rhio[tw] OR regional health information organization[tw] OR hl7[tw] OR health level seven[tw] OR "unified medical language system"[MeSH Major Topic] OR umls[tw] OR loinc[tw] OR rxnorm[tw] OR snomed[tw] OR icd9 cm[ti] OR icd 9 cm[ti] OR icd10[ti] OR icd 10[ti] OR metathesaurus[tw] OR patient card[tw] OR patient cards[tw] OR health card[tw] OR health cards[tw] OR electronic health data[tw] OR personal health data[tw] OR personal health record[tw] OR personal health records[tw] OR "health records, personal"[MeSH Major Topic] OR "health records, personal"[MeSH Major Topic] OR ehealth[tw] OR e-health[tw] OR "medical informatics applications"[MeSH Terms] OR "medical informatics applications"[MeSH Terms] OR "medical records systems, computerized"[MeSH Terms] OR "medical records systems, computerized"[MeSH Terms] OR computerized patient medical records[tw] OR automated medical record system[tw] OR automated medical record systems[tw] OR automated medical records system[tw] OR automated medical records systems[tw] OR computerized medical record[tw] OR computerized medical records[tw] OR computerized patient records[tw] OR computerized patient record[tw] OR computerized patient medical record[tw] OR electronic health record[tw] OR electronic health records[tw] OR "electronic health records"[MeSH Major Topic] OR "electronic health records"[MeSH Major Topic] OR electronic patient record[tw] OR electronic patient records[tw] OR electronic medical record[tw] OR electronic medical records[tw] OR electronic healthcare records[tw] OR electronic healthcare record[tw] OR electronic health care record[tw] OR electronic health care records[tw] OR "archives"[MeSH Major Topic] OR ehr[tw] OR ehrs[tw] OR phr[tw] OR phrs[tw] OR emr[tw] OR emr[tw] OR "health information systems"[MeSH Major Topic]) AND (medical record[ti] OR "medical records"[MeSH Terms] OR medical records[ti] OR patient record[ti] OR patient records[ti] OR patient health record[ti] OR patient health records[ti] OR "patient identification systems"[MeSH Terms] OR "patient identification systems"[MeSH Terms] OR healthcare record[ti] OR healthcare records[ti] OR health care record[ti] OR health care records[ti] OR health record[ti] OR health records[ti] OR hospital information system[tw] OR hospital information systems[tw] OR umae[ti] OR "attitude to computers"[MeSH Terms] OR medical informatics[ti])) OR (("medical records systems, computerized"[MeSH Major Topic] OR "medical records systems, computerized"[MeSH Terms] OR computerized patient medical record[tw] OR computerized patient medical records[tw] OR automated medical record system[tw] OR automated medical record systems[tw] OR automated medical records system[tw] OR automated medical records systems[tw] OR computerized medical record[tw] OR computerized medical records[tw] OR computerized patient records[tw] OR computerized patient record[tw] OR electronic health record[tw] OR electronic health records[tw] OR electronic patient record[tw] OR electronic patient records[tw] OR electronic medical record[tw] OR electronic medical records[tw] OR electronic healthcare records[tw] OR electronic healthcare record[tw] OR electronic health care record[tw] OR electronic health care records[tw] OR "unified medical language system"[MeSH Major Topic] OR unified medical language system[tw] OR umls[tw] OR loinc[tw] OR rxnorm[tw] OR snomed[tw] OR icd9 cm[ti] OR icd 9 cm[ti] OR icd10[ti] OR icd 10[ti] OR Metathesaurus[tw] OR ehr[tw] OR ehrs[tw] OR phr[tw] OR phrs[tw] OR emr[tw] OR emrs[tw] OR meaningful use[tiab] OR meaningful use[tw] OR "meaningful use"[MeSH Major Topic]) AND ("J AHIMA"[Journal] OR "J Am Med Inform Assoc"[Journal] OR "AMIA Annu Symp Proc"[Journal] OR "Health Data Manag"[Journal] OR "Int J Med Inform"[Journal] OR "Yearb Med Inform"[Journal] OR "Telemed J E Health"[Journal] OR "Stud Health Technol Inform"[Journal])))
#2	("Registries"[mh] OR register*[tiab] OR registr*[tiab])
#1	("Databases as Topic"[mh] OR database*[tiab] OR "health care databases"[tiab] OR "healthcare databases"[tiab] OR "health care database"[tiab] OR "healthcare database"[tiab] OR "healthcare data"[tiab] OR "health care data"[tiab] OR "national database"[tiab])

Date of search: 6 June 2013

Webappendix 2: Definitions of types of routinely collected health data

Routinely collected health data	The blanket term for any health information collected routinely and deposited in a database. While research is conducted using these data later, the data are not collected with the specific research question in mind. [1]
EMR/EHR	Electronic database of information collected from a patients chart. The data is collected for clinical purposes, for example during consultation in primary care or at the bedside, and deposited into a database for later use in research or for other purposes.
Health administrative data	Health administrative data are defined as information passively collected, often by government and health care providers, for the administrative purpose of managing the health care of patients. [2]<br>*Examples:* government health databases, physician billing databases, hospitalization databases, medication prescription databases, insurance company databases
Registry data (patient/disease)	Patient registries: "an organized system that uses observational study methods to collect uniform data (clinical and other) to evaluate specified outcomes for a population defined by a particular disease, condition, or exposure, and that serves one or more predetermined scientific, clinical, or policy purposes". [14]<br>*Examples:* disease registries, cancer registries
Registry data (device)	Device registries - as above, but tracking the use of devices.
Other Administrative Data	These data are similar to health administrative data but are not restricted to medical or health information. However, these data may later be used for health research. [2]<br>*Examples:* birth/death registries, immigration databases, registered persons databases
Mobile health data	Data collected by mobile devices such as smartphones or wearable technology and used later for research.

EMR: Electronic Medical Record; EHR: Electronic Health Record

Webappendix 3: Evaluation of reporting items: Details

Basic classification of study design and indication of the use of routine data

Item	Description
[S1]	**Is the study's design indicated with a commonly used term in the title or the abstract?**<br><br>We accepted any term for study designs (such as "cohort study" or "case-control study") used in typical study classification schemes [19].<br><br>*Examples are given in STROBE checklist (item 1a)* [22]
[R1]	**Is the use of routinely collected data or registry data clearly mentioned in the title or the abstract using common terms?**<br><br>We evaluated whether information in the title or abstract allows a reader or a database search engine to clearly recognize the use of routinely collected or registry data.<br><br>***Examples:***<br>▪ *"[...] by using a national Veterans Administration database [...]"* [23]<br>▪ *"Data included secondary enrollment and demographic data from Washington Dental Service (WDS) and Group Health Cooperative (GH), clinical data from GH, and dental-utilization data from WDS claims during 2002-2006"* [24]<br>▪ *"Data from central cancer registries in the National Program of Cancer Registries (NPCR) and Surveillance, Epidemiology, and End Results (SEER) programs [...] were analyzed.* [25]

STROBE: STrengthening the Reporting of OBservational studies in Epidemiology

Details on population, exposures or interventions and outcomes

Item	Description
[S2]	**Are the selection criteria for the analyzed participants clearly described?**<br>This was deemed adequate when the study participant selection was reported in a way that it would be clear to whom the results directly apply and for whom they would not be applicable.<br>*Examples are given in STROBE checklist (item 6)* [22]
[S3]	**Are all interventions/exposures of interest clearly described?**<br>We deemed an exposure or intervention (or risk factor, predictor, effect modifier etc.) sufficiently described when the provided details would allow the application of the intervention or the measurement of exposure (or risk factor etc.) in practice. The reader should know precisely which action (*e.g.,* prescription of a certain dose of a drug) or exposure is being assessed in the study [18].<br>*Examples are given in STROBE checklist (item 7)* [22]
[S4]	**Are all outcomes of interest clearly described?**<br>The outcome description was deemed adequate if it was equivalent to an outcome description in a planned prospective study designed to specifically investigate the issue (regardless whether such study would be interventional or observational, feasible or not) and if the detail given was sufficient for others to replicate the study. We did not assess if broad or specific outcomes were used, but we assessed if the reporting clearly defined the outcome and how it was measured and defined. For example, we deemed it insufficient when authors reported “we analyzed effects on hypertension” without giving a definition of hypertension (e.g., defined by more than one prescription of an antihypertensive drug within 6 months); or when authors say “we evaluated effects on mortality” without stating whether all-cause or cause-specific mortality has been investigated and without reporting the time-frame (e.g., in-hospital mortality or 30-day-after discharge mortality).<br>*Examples are given in STROBE checklist (item 7)* [22]

STROBE: STrengthening the Reporting of OBservational studies in Epidemiology

Codes and classification algorithms and other basic requirements for replication of analyses.

Item	Description
[R2]	**Is the coding/classification of patients clearly described with sufficient details?**<br><br>We deemed reporting adequate when the description of the coding or classification algorithm was sufficiently clear to allow replication of the analysis.<br>***Examples:***<br>▪ *"[...] we identified all patients aged ≥ 18 years who underwent a colon resection for a diagnosis of colon cancer [International Classification of Diseases, Ninth Revision, Clinical Modification (ICD-9-CM) codes 153.0 – 153.4, 153.6 – 153.9, and 230.3] between October 1, 2008 and October 31, 2009 during an elective admission. [...] we excluded patients who required a total or transverse colectomy or had concurrent procedures (hepatic, small bowel, pancreas, or bladder resection), were pregnant, or had evidence of metastatic disease. "* [26]<br>▪ *"We identified children younger than 18 years discharged from a PHIS hospital between January 1, 2001, and June 30, 2011, with an International Classification of Diseases, Ninth Revision, Clinical Modification (ICD-9-CM) discharge diagnosis code for TBI (Figure 1). [...]To be included, patients were required to have either ICD-9-CM procedure code 96.72, which represents continuous invasive mechanical ventilation for 96 consecutive hours or more, or mortality in the first 4 days after admission. We calculated the Injury Severity Score (ISS, or specifically, ICD/ISS) and maximum Abbreviated Injury Scale (AIS) body region scores from ICD-9-CM diagnosis codes [...] we excluded patients with maximum head body region AIS scores of less than 3 (serious), patients with missing head AIS scores, patients with missing disposition, and patients with subsequent admissions[...]"* [27]
[R3]	**Is the coding/classification of the interventions/exposures clearly described with sufficient details?**<br><br>We deemed reporting adequate when the description of the coding or classification algorithm was sufficiently clear to allow replication of the analysis.<br>***Examples:***<br>▪ *"Patients were then categorized as having undergone a laparoscopic (ICD-9-CM codes 17.3, 17.31 – 17.39) or an open (ICD-9-CM codes 45.7, 45.71 – 45.79) colon resection. [...] cases with evidence of a procedure converted to open (ICD-9-CM codes V64.40, V64.41) were considered to have had a laparoscopic procedure and counted in this group."* [26]<br>▪ *"The incident OAD monotherapy categories were metformin, sulfonylurea, and rosiglitazone. Combination therapies and insulin users during the baseline period were excluded [...]"* [23]
[R4]	**Is the coding/classification of the outcomes clearly described with sufficient details?**<br><br>We deemed reporting adequate when the description of the coding or classification algorithm was sufficiently clear to allow replication of the analysis. We deemed it unnecessary for replication that all-cause mortality is operationalized with a specific code, because this outcome is typically clear.<br><br>***Examples:***<br>▪ *"The primary outcome was ICP monitoring, defined using Clinical Transaction Classification codes or ICD-9-CM procedure codes (see the footnotes to Table 2). [...] We defined poor outcome as hospital mortality or placement of a new tracheostomy tube (ICD-9-CM procedure codes 31.1, 31.2x, or 31.74) and a new gastrostomy tube (ICD-9-CM procedure codes 43.1x, 46.32, or 46.39) during the hospitalization."* [27]<br>▪ *"The primary end point was a composite of a GFR event or reaching ESRD. [...]*<br>*"A GFR event was defined as a persistent 25% or greater decline from the baseline eGFR.*

[...]" GFR event [...] 25% decrease in GFR (~30 mL/min/1.73 m2) noted on the first of 2 outpatient laboratory values. Requires change to be present on 2 outpatient GFR calculations between 3 and 12 months apart [...]" [23]

[S5] **Are the independent variables in analytic models**

a) listed (or are the strategies used to create models reported)?

We deemed reporting adequate when all analyzed variables (e.g., age, body weight, smoking) were listed.

b) described in sufficient detail (including categorization) to replicate the study?

We deemed reporting adequate when details were provided on how the variables were included in the statistical models (e.g., age and body weight both as continuous variable and smoking as categorical variable such as "never smokers", "previous smokers", "smoking 1 to 10 cigarettes daily", and "smoking more than 11 cigarettes daily").

Examples are given in STROBE checklist (items 7 and 11) [22]

[R5] **Are the characteristics of the analyzed datasets clearly described, including (1) covered time period, (2) location, (3) setting and other potentially important factors?**

We deemed that reporting was adequate when the covered time period, geographic location, care setting, and other potentially important factors (for example essential details about type of data used; decision on a case-by-case basis) were reported.

Examples:

- *"We conducted a retrospective cohort study of diabetic patients seen within the VHA system between 1 October 2001 and 30 September 2008. The cohort was constructed using national VHA databases from the Decision Support Services, which contain prescriptions data and laboratory results. The primary source of the prescription data was the Veterans Health Information System and Technology Architecture (VistA) and included inpatient and outpatient prescriptions dispensed by a VHA Pharmacy or a Consolidated Mail Outpatient Pharmacy. The VHA national medical data sets contain electronically captured patient demographics and diagnostic and procedure information from inpatient and outpatient encounters, coded according to the International Classification of Diseases, Ninth Revision; Clinical Modification (ICD9-CM). Data on vital signs (blood pressure, weight, and height) and vital status were obtained from the VHA Corporate Data Warehouse and Vital Status master "* [23]
- *"This study was conducted using linked data from Washington Dental Service (WDS) and Group Health Cooperative (GH). [...]WDS is a founding member of the Delta Dental Plans Association delivering dental care to more than two million people through employer-sponsored programs. GH is a nonprofit health care system that coordinates care and coverage to more than half a million residents of Washington state and Idaho. Secondary enrollment data from WDS and GH, clinical (laboratory, pharmacy and diagnosis) data from GH, and claims data from WDS were obtained for enrollees continuously and dually insured between January 1, 2002 and December 31, 2006."* [24]

STROBE: STrengthening the Reporting of OBservational studies in Epidemiology

RCD specifics

Item	Description
[R6]	**Are the methods of linkage of databases clearly described (if applicable)?**<br><br>***Examples:***<br>▪ *"[…] we selected only those patients who had a unique combination of date of birth, gender and postal code in order to allow valid linkage with the population register (and, through the population register, with the income and cause of death registers). We linked our patients with the income register for the period 2002 - 2004 and selected only those patients for whom information on income in at least one of these 3 years was available."* [28]<br>▪ *"Linkage of recipients to registers in this study was done by their personal identification number (PIN), which is a unique 10-digit code assigned to all permanent residents of Denmark."* [29]
[R7]	**Are issues of data sharing clearly addressed, i.e. whether the dataset is publicly available (or shared on request)?**<br><br>We accepted any statement regardless of how detailed it was.<br>***Examples:***<br>▪ *"The SEER public use database 1973 to 2007 (Version April 2010) was used for this analysis."* [30]<br>▪ *"The data used are available for research projects from the cancer registry of Norway and the Norwegian Institute of Public Health, who are the legal administrators of the data. The data can, however, not be shared directly with other researchers."* [31]<br>▪ *"Data for the NIS are collected at the state level from hospital discharge records, reported to the Agency for Healthcare Research and Quality, and made publicly available via the Healthcare Cost and Utilization Project."* [26]
[R8]	**Is the validation of classification algorithms used for patients, interventions/ outcomes/ exposures described (if applicable)?**<br><br>***Examples:***<br>▪ *"The quality of the data is high due to thorough and uniform training of the registration clerks and computerized consistency checks at regional and national levels. Completeness is estimated to be at least 95% [REFERENCE], except for CLL, a diagnosis regularly made without tissue specimens [REFERENCE]"* [32]<br>▪ *"This definition of a diabetes case is consistent with the definition validated in prior research [REFERENCE]."* [33]

References:

22. Vandenbroucke JP, von Elm E, Altman DG, et al. Strengthening the Reporting of Observational Studies in Epidemiology (STROBE): explanation and elaboration. PLoS medicine 2007;**4**(10):e297.
23. Hung AM, Roumie CL, Greevy RA, et al. Comparative effectiveness of incident oral antidiabetic drugs on kidney function. Kidney international 2012;**81**(7):698-706.
24. Ng-Mak DS, Chen YT, Ho TW, et al. Results of a 2-year retrospective cohort study of newly prescribed triptan users in European nationwide practice databases. Cephalalgia : an international journal of headache 2012;**32**(12):875-87.
25. Duong LM, McCarthy BJ, McLendon RE, et al. Descriptive epidemiology of malignant and nonmalignant primary spinal cord, spinal meninges, and cauda equina tumors, United States, 2004-2007. Cancer 2012;**118**(17):4220-7.
26. Fox JP, Desai MM, Krumholz HM, et al. Hospital-level outcomes associated with laparoscopic colectomy for cancer in the minimally invasive era. Journal of gastrointestinal surgery : official journal of the Society for Surgery of the Alimentary Tract 2012;**16**(11):2112-9.
27. Bennett TD, Riva-Cambrin J, Keenan HT, et al. Variation in intracranial pressure monitoring and outcomes in pediatric traumatic brain injury. Archives of pediatrics & adolescent medicine 2012;**166**(7):641-7.
28. Stirbu I, Looman C, Nijhof GJ, et al. Income inequalities in case death of ischaemic heart disease in the Netherlands: a national record-linked study. Journal of epidemiology and community health 2012;**66**(12):1159-66.
29. Just SA, Grau K, Georgsen J, et al. Long-term follow-up among Danish transfusion recipients identified in the national hepatitis C lookback. Transfusion 2012;**52**(3):582-8.
30. Bohanes P, Yang D, Chhibar RS, et al. Influence of sex on the survival of patients with esophageal cancer. Journal of clinical oncology : official journal of the American Society of Clinical Oncology 2012;**30**(18):2265-72.
31. Weedon-Fekjaer H, Bakken K, Vatten LJ, et al. Understanding recent trends in incidence of invasive breast cancer in Norway: age-period-cohort analysis based on registry data on mammography screening and hormone treatment use. BMJ (Clinical research ed) 2012;**344**:e299.
32. van de Schans SA, Issa DE, Visser O, et al. Diverging trends in incidence and mortality, and improved survival of non-Hodgkin's lymphoma, in the Netherlands, 1989-2007. Annals of oncology : official journal of the European Society for Medical Oncology / ESMO 2012;**23**(1):171-82.
33. Chaudhari M, Hubbard R, Reid RJ, et al. Evaluating components of dental care utilization among adults with diabetes and matched controls via hurdle models. BMC oral health 2012;**12**:20.
